# A multicenter hemodynamics–based nomogram predicting incomplete occlusion of intracranial aneurysms treated with pipeline embolization device

**DOI:** 10.3389/fneur.2026.1756374

**Published:** 2026-02-09

**Authors:** Yawen Zhao, Li Bao, Shuang He, Yunfeng Zhang

**Affiliations:** 1 Department of Stroke Center, Affiliated Hospital of Nantong University, Nantong, China; 2 Department of Cardiology, Affiliated Hospital of Nantong University, Nantong, China

**Keywords:** hemodynamics, intracranial aneurysms, LASSO, nomogram, pipeline embolization device

## Abstract

**Background:**

This multicenter study aimed to develop and validate a hemodynamics-based nomogram for predicting incomplete occlusion (ICO) of intracranial aneurysms (IAs) after pipeline embolization device (PED) treatment.

**Methods:**

426 IAs from 362 patients were analyzed and divided into a training set (*n* = 298) and a validation set (*n* = 128). Morphological and hemodynamic parameters of the IAs were calculated using AneuFlow Pro. Independent predictors of ICO were identified using least absolute shrinkage and selection operator (LASSO) regression and logistic regression to develop a predictive nomogram. The nomogram's performance was evaluated using area under the curve (AUC), calibration curves, and decision curve analysis (DCA).

**Results:**

The aneurysm occlusion rate of the overall cohort was 79.8% with a median angiographic follow-up time of 199 days. No significant differences were observed in patient and aneurysm characteristics between the training and validation sets. Through LASSO and logistic regression analyses, we identified smoking (OR = 0.32, 95% CI 0.14–0.68, *p* = 0.005), flow complexity (OR = 3.03, 95% CI 1.58–5.89, *p* < 0.001), device migration (OR = 11.03, 95% CI 1.51–105.55, *p* = 0.021), poor wall apposition (OR = 3.21, 95% CI 1.37–7.53, *p* = 0.007), aneurysm angle (OR = 3.46, 95% CI 1.79–6.93, *p* < 0.001), and low wall shear stress area ratio (LSAR; OR = 2.78, 95% CI 1.46–5.50, *p* = 0.002) as independent predictors of ICO. A nomogram developed based on these factors showed an AUC of 0.785 (95% CI 0.719–0.850) in the training set and 0.809 (95% CI 0.695–0.923) in the validation set, demonstrating consistent calibration and excellent clinical use.

**Conclusion:**

The hemodynamics-based nomogram developed in this study effectively predicted ICO of IAs after PED treatment.

## Introduction

In recent years, pipeline embolization device (PED) have been widely used for endovascular treatment of complex or large intracranial aneurysms (IAs), proving to be both safe and effective (1–3). However, approximately 20% of IAs may exhibit persistent incomplete occlusion (ICO) for over 12 months (4–6), presenting a significant challenge in the use of PED. ICO maintains the risk of aneurysm recurrence and rupture. Therefore, predicting and promoting complete occlusion (CO) of IAs treated with PED is crucial for preventing adverse events.

Previous studies have highlighted clinical features, including age, smoking, and specific aneurysm anatomical parameters, as predictors of PED treatment failure in IAs (7–9). However, the impact of hemodynamics, which play a crucial role in IA growth and rupture, on IA occlusion after PED treatment remains unclear. In this multicenter study, a preoperative hemodynamic analysis based on digital subtraction angiography (DSA) were performed to identify independent predictors of ICO of IAs treated with PED. We developed and validated the first nomogram predicting ICO after PED treatment. This nomogram, which incorporates preoperative hemodynamic parameters, could offer a foundation for the timely identification of high-risk ICO IAs and the implementation of optimized treatment strategies (e.g., adjunctive coil embolization and regular follow-up), thereby enhancing treatment personalization, reducing the risk of failure, and improving the prognosis of IAs.

## Methods

### Study design

This multicenter retrospective study analyzed consecutive patients with IAs treated with PED between Jan 2023 and November 2024 from four centers: the Stroke Center at the West Campus of the Affiliated Hospital of Nantong University, the Neurointerventional Center at the East Campus of the Affiliated Hospital of Nantong University, Jianghai Hospital of Sutong Technology Industrial Park and Rugao Bo'ai Hospital. Inclusion criteria were as follows: (1) age 18–85 years; (2) IAs initially treated with PED Flex. The exclusion criteria were as follows: (1) patients with arteriovenous malformations or fistulas; (2) IAs previously treated with endovascular or neurosurgical treatment; (3) poor image quality or lack of angiographic follow-up data; (4) patients with a history of malignancy. This multicenter study was approved by the Institutional Review Board of the Affiliated Hospital of Nantong University (2021-Q094-01), and informed consent was waived owing to the retrospective nature of the study.

The primary outcome of this study was the ICO rate of IAs at the first follow-up DSA (FU-DSA) after PED treatment. ICO was defined as residual contrast filling in the aneurysm on FU-DSA, corresponding to O'Kelly-Marotta grading of A, B, or C (10). Patient and aneurysm characteristics, preoperative hemodynamic parameters and procedural details were recorded. The variable “Smoking” was defined as having a smoking history of more than 5 years or currently smoking.

### Treatment protocol

Patients were premedicated with dual antiplatelet therapy with 100 mg aspirin and 75 mg clopidogrel daily 10 days before PED implantation. Platelet function was assessed using thromboelastography 5 days before the procedure. For patients with aneurysmal subarachnoid hemorrhage undergoing emergency PED implantation, we administered intravenous tirofiban (0.1 μg/kg·min) within 24 h post-PED implantation, followed by overlapping and subsequent alternation of dual antiplatelet therapy. Platelet function testing was performed 5 days postoperatively. Patients with an inhibition rate below 30% were considered hyporesponsive to clopidogrel and were switched to ticagrelor at a dose of 90 mg twice daily. Dual antiplatelet therapy was continued for at least 6 months postoperatively, while single antiplatelet therapy was continued indefinitely.

Patients received general anesthesia and heparin anticoagulation throughout the procedure. A standard combination of a 6-Fr long sheath (Ballast, Balt, USA), an intermediate catheter (Zenith, China), a microcatheter (Phenom 27, Medtronic, USA), and a microwire (Synchro 14, Stryker, USA) was utilized under a coaxial system. The decision to use adjunctive coil embolization was made based on the operator's judgment regarding the risk of aneurysm rupture and recurrence. Loose packing was selected for coil embolization, adhering to a standard filler density of 12% in this study (11). The migration and wall apposition of the PED was evaluated using Vaso-CT (Philips). FU-DSA was conducted to assess PED treatment efficacy after at least 6 months. Device migration was defined as any noticeable displacement of one or both ends of the PED, observed on immediate postoperative or FU-DSA compared to the intraoperative release site (12). Poor wall apposition was defined as the presence of any detectable gap between the stent and the vessel wall after repeated massage with the “J”-shaped microwire, as identified by immediate VasoCT. If necessary, post-balloon angioplasty was performed to facilitate wall apposition. All angiographic results were evaluated by two independent neuroradiologists from the imaging core laboratory who were blinded to both clinical characteristics and outcomes. Any discrepancies in the evaluations were resolved by a senior radiologist.

### Computational fluid dynamics modeling

The preoperative 3D DSA segmentation DICOM data of each IA was imported into AneuFlow Pro (ArteryFlow, China) for automatic segmentation and mesh generation, creating a 3D model of the aneurysm and the parent artery that accurately reconstructed the morphology of the aneurysm region. The maximum mesh size is set at 0.1 mm with three layers of wall prism elements. The aneurysm model comprises 1.8–2.3 million tetrahedral elements post-meshing. Blood was assumed to be an incompressible, laminar Newtonian fluid, with a density of 1,056 kg/m3 and a dynamic viscosity of 0.0035 N·s/m^2^. A rigid-wall and no-slip boundary condition was implemented at each vessel wall with the heat transfer and the compressibility effects neglected. Incompressible Navier-Stokes equations were solved numerically under pulsatile flow conditions. The inlet pulsatile velocity waveform was obtained from Transcranial Doppler ultrasound measurement on a normal subject and a published mean flow rate of 4.6 ml/s was used as the internal carotid artery inlet boundary condition. The mass flow rate through each outlet artery was proportional to the cube of its diameter. Three pulsatile cycles were simulated to ensure that numeric stability was achieved, and the simulation results from the last cycle were taken as the output. All the following parameters were the average values of the last cycle or calculated based on the results of the entire last cycle (13, 14).

### Morphologic and hemodynamic analysis

The morphological parameters were calculated automatically by the AneuFlow Pro software based on the established 3D vascular model, including the maximum height of the aneurysm, the middle diameter of the aneurysm, the neck diameter of the aneurysm, the diameter of the parent artery, the vertical height of the aneurysm, the surface area of the aneurysm, the volume of the aneurysm, the inflow angle of the aneurysm, the aneurysm angle, the parent artery angle, the size ratio, the aspect ratio, the undulation index, the non-sphericity index, the ellipticity index and the body-neck ratio. The morphological parameters were shown schematically in Supplementary Figure 1.

Based on the simulated flow fields, following hemodynamic parameters were calculated: mean wall shear stress (WSS), mean normalized WSS, mean parent vessel WSS, mean maximum WSS, mean minimum WSS, high WSS area ratio, low WSS area ratio (LSAR), mean oscillatory shear index, mean maximum OSI, WSS gradient, gradient oscillatory number, and relative residence time. LSAR was defined as the area ratio of the aneurysm wall where the WSS is less than 10% of the mean parent vessel WSS (15).

In addition to these quantitative variables, some qualitative variables related to blood flow (such as flow complexity and inflow jet) were assessed using the method proposed by Cebral et al. (16).

### Development and validation of the nomogram

We randomly divided the entire cohort into a nomogram development set and a validation set in a 7:3 ratio. The training cohort was used to develop the nomogram. The least absolute shrinkage and selection operator (LASSO) method, which is suitable for regression with high-dimensional data, was used to select the most useful predictive features from the original dataset and model complexity regularization, thereby minimizing potential multicollinearity among variables and reducing the risk of overfitting (17). Combining the nonzero coefficient variables selected from LASSO, a multivariate backward stepwise logistic regression was conducted to identify independent predictive factors and to develop a nomogram. The performance of the nomogram was evaluated through internal validation (training cohort) and external validation (validation cohort). The discriminative ability of the nomogram was evaluated using the area under the receiver operating characteristic (ROC) curve (AUC). Calibration curves were plotted based on 1,000 bootstrap resampling to evaluate the calibration of the nomogram. Decision curve analysis (DCA) was primarily used to assess the clinical value of the nomogram by calculating the net benefit at different threshold probabilities.

### Statistical analysis

Statistical analysis was performed using R Studio (version 4.4.2). The normality of continuous variables was assessed using the Kolmogorov-Smirnov test. Continuous variables were presented as median (IQR), while categorical variables were presented as frequency (percentage). Comparisons of continuous variables were conducted using independent *t*-tests or Mann–Whitney *U* tests, while comparisons of categorical variables were analyzed using χ^2^ tests or Fisher's exact tests. A 2-tailed *P* value < 0.05 was considered statistically significant.

Independent predictive factors were identified through LASSO selection and logistic regression, and a nomogram was constructed based on these factors. Multicollinearity was assessed using correlation matrices and the variance inflation factor. The predictive performance of the nomogram was evaluated in both the training and validation sets using ROC curve, calibration curve, and DCA.

## Results

### Patient and aneurysm characteristics

A total of 426 IAs from 362 patients who received PED treatment were included in the final analysis. Among these, 72.7% (263/362) were female, and the median age was 63 (IQR 56–70) years. 61 patients received a single PED treating two or more adjacent aneurysms. All aneurysms were covered by a single PED. At the 6-month follow-up, spontaneous mild asymptomatic delayed migration of the PED still covering the aneurysm, was observed in eight patients, which was potentially associated with relatively undersized stent diameter in six cases and a significant proximal-to-distal diameter gradient of the parent artery in two cases. Forty nine PED treatment exhibited poor wall apposition (7 occurred at the aneurysm neck), despite repeated massage with the “J”-shaped microwire. One patient developed symptomatic acute in-stent thrombosis probably related to intimal injury caused by microwire massage and post-balloon angioplasty after PED implantation, but following arterial thrombolysis, in-stent recanalization was achieved with no remaining neurological deficits. Three patients developed symptomatic intracranial hemorrhage, one due to intraoperative aneurysm rupture and two due to hyperperfusion following decompression of giant aneurysms on postoperative day 1. The hematomas were absorbed without residual neurological deficits.

**Figure 1:**
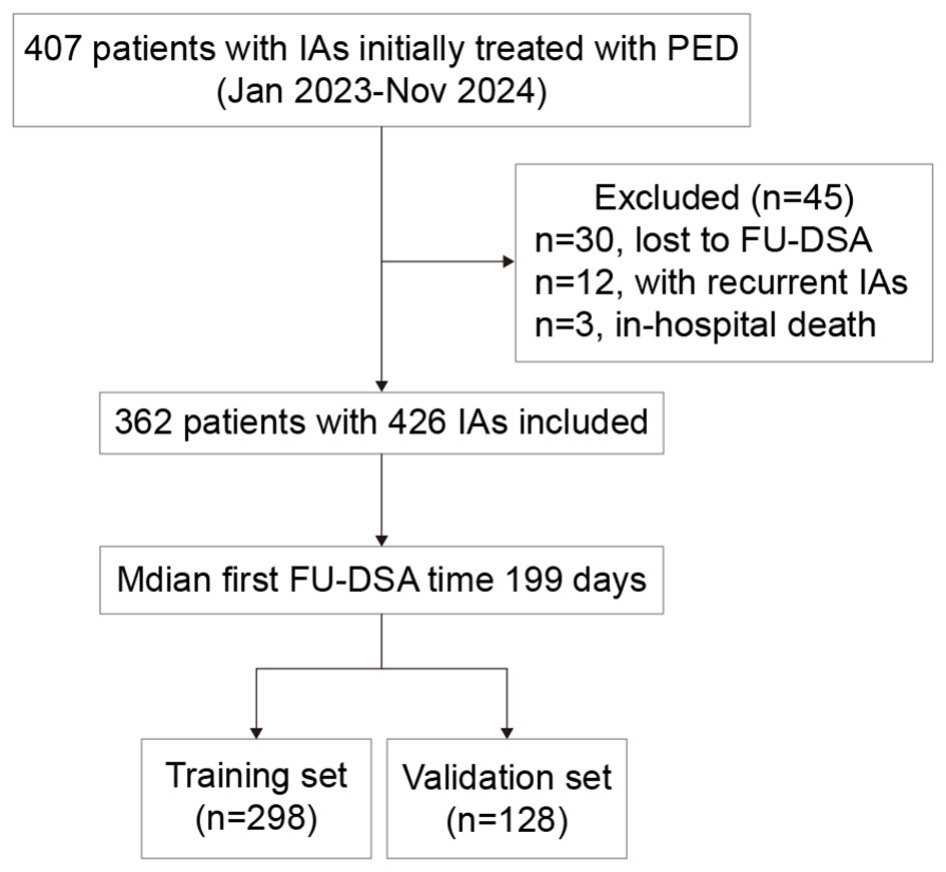
Study flowchart. IA, intracranial aneurysm; PED, flow diverter; FU-DSA, follow up digital subtraction angiography.

The median FU-DSA time for the whole cohort was 199 (191–244) days, and the CO rate was 79.8% (340/426). All aneurysms were randomly assigned to the training cohort (*n* = 298) and the validation cohort (*n* = 128) in a 7:3 ratio. The study flowchart was shown in Figure 1. There was no significant difference in the CO rate between the training set and the validation set (78.5 vs 82.8%, *p* = 0.38). As shown in Table 1, there were no statistically significant differences in variables between the training and validation cohorts, confirming that both cohorts had similar baseline characteristics.

**Table 1:** Clinical characteristics and morphology and hemodynamic parameters in the training and validation cohorts.

**Variables**	**Training cohort (*n* = 298)**	**Validation cohort (*n* = 128)**	***P***	**Variables**	**Training cohort (*n* = 298)**	**Validation cohort (*n* = 128)**	***P***
Age (years)	64 (56–70)	62.5 (56–70)	0.94	Inflow angle (°)	88.2 (64.3–112.0)	89.5 (65.3–113.9)	0.51
Female	214 (71.8)	99 (77.3)	0.29	Aneurysm angle (°)	86.2 (58.4–111.7)	95.8 (63.0–115.0)	0.36
Hypertension	141 (47.3)	54 (42.2)	0.39	Parent artery angle (°)	2.9 (-10.9–15.8)	3.7 (-11.2–17.6)	0.66
Diabetes	39 (13.1)	15 (11.7)	0.82	*H*_max_ (mm)	4.4 (3.1–6.0)	4.0 (3.0–5.7)	0.24
Hyperlipidemia	85 (28.5)	34 (26.6)	0.77	*D*_middle_ (mm)	4.9 (3.7–6.5)	4.5 (3.6–6.1)	0.31
Coronary heart disease	16 (5.4)	6 (4.7)	0.96	*D*_neck_ (mm)	4.2 (3.2–5.3)	4.0 (3.2–5.3	0.55
Atherosclerosis	110 (36.9)	43 (33.6)	0.59	*D*_vessel_ (mm)	3.1 (2.5–3.7)	3.1 (2.5–3.6)	0.80
Smoking	90 (30.2)	32 (25.0)	0.33	H (mm)	3.7 (2.7–5.2)	3.3 (2.5–4.9)	0.20
Alcohol abuse	40 (13.4)	15 (11.7)	0.75	Aneurysm surface area (mm^2^)	58.2 (30.3–115.5)	48.6 (27.7–05.9)	0.21
mRS scores ≥2	60 (20.1)	37 (28.9)	0.06	Aneurysm volume (mm^3^)	46.7 (18.0–118.4)	35.7 (16.5–102.6)	0.24
History of multiple aneurysms	107 (35.9)	41 (32.0)	0.51	Aspect ratio	0.8 (0.7–1.1)	0.8 (0.7–1.0)	0.31
Aneurysm Presentation			0.93	Uncertainty Index	0.05 (0.03–0.12)	0.05 (0.03–0.11)	0.06
Asymptomatic	62 (20.8)	28 (21.9)		Size ratio	1.5 (1.0–2.1)	1.4 (0.9–2.0)	0.55
Headache	180 (60.4)	76 (59.4)		Ellipticity Index	0.1 (0.06–0.14)	0.1 (0.06–0.14)	0.34
Tinnitus	2 (0.7)	2 (1.6)		Non-sphericity index	0.1 (0.07–0.20)	0.1 (0.07–0.19)	0.30
Vertigo	8 (2.7)	5 (3.9)		Body-neck ratio	1.1 (1.0–1.2)	1.1 (1.0–1.2)	0.49
Nerve Palsy	12 (4.0)	5 (3.9)		WSS (Pa)	4.8 (2.6–7.7)	5.6 (2.9–8.0)	0.15
TIA/ischemia	28 (9.4)	9 (7.0)		NWSS (Pa)	0.6 (0.5–0.9)	0.7 (0.5–0.9)	0.52
Ruptured	6 (2.0)	3 (2.3)		WSS_vessel_ (Pa)	7.8 (5.0–11.3)	7.7 (6.0–11.5)	0.48
Bifurcation	20 (6.7)	7 (5.5)	0.79	WSS_max_ (Pa)	21.2 (12.7–32.1)	20.9(13.7–31.5)	0.53
Aneurysm location			0.61	WSS_min_ (Pa)	0.4 (0.2–0.9)	0.5 (0.2–1.0)	0.18
ICA	270 (90.6)	121 (94.5)		HSAR (%)	20.7(10.0–38.1)	25.0 (10.8–46.2)	0.37
MCA	4 (1.3)	1 (0.8)		LSAR (%)	1.5 (0.0–7.9)	1.1 (0.0–7.1)	0.40
ACA	4 (1.3)	1 (0.8)		OSI (^*^10^−2^)	0.6 (0.4–1.1)	0.5 (0.3–1.0)	0.49
VBA	20 (6.7)	5 (3.9)		OSI_max_ (^*^10^−2^)	16.9 (9.8–28.8)	15.1 (9.1–26.3)	0.31
Aneurysm morphology			0.72	WSSG (Pa/mm)	5.2 (2.8–9.0)	6.4 (3.5–11.3)	0.07
Saccular	264 (88.6)	116 (90.6)		GON	0.07 (0.04–0.10)	0.06 (0.04–0.10)	0.62
Fusiform	12 (4.0)	6 (4.7)		RRT (Pa^−1^)	0.4 (0.2–0.9)	0.3 (0.2–0.6)	0.15
Dissecting	20 (6.7)	5 (3.9)		Adjunctive coiling	22 (7.4)	9 (7.0)	>0.99
Blister	2 (0.7)	1 (0.8)		Device migration	6 (2.0)	2 (1.6)	>0.99
Incorporating branches from sac	68 (22.8)	25 (19.5)	0.53	Poor wall apposition	36 (12.1)	13 (10.2)	0.69
Flow complexity	109 (36.6)	45 (35.2)	0.87	Immediate postoperative occlusion	4 (1.3)	2 (1.6)	>0.99
Inflow jets	12 (4.0)	6 (4.7)	0.96	FU-DSA time (days)	198 (192–244)	199 (191–231.5)	0.81

Values are shown as median (IQR) or frequency (%).

mRS, modified rankin scale; ICA, internal carotid artery; MCA, middle cerebral artery; ACA, anterior cerebral artery; VBA, vertebrobasilar artery; H_max_, maximum height of the aneurysm; D_middle_, middle diameter of the aneurysm; D_nec_k, neck diameter of the aneurysm; D_vessel_, diameter of the parent artery; H: vertical height of the aneurysm; WSS, mean wall shear stress; NWSS, mean normalized WSS; WSS_vessel_, mean parent vessel WSS; WSS_max_, mean maximum WSS; WSS_min_, mean minimum WSS; HSAR, high WSS area ratio; LSAR, low WSS area ratio; OSI, mean oscillatory shear index; OSI_max_, mean maximum OSI; WSSG, WSS gradient; GON, gradient oscillatory number; RRT, relative residence time; FU-DSA, follow up- digital subtraction angiography.

### Variable selection and nomogram development

**Figure 2:**
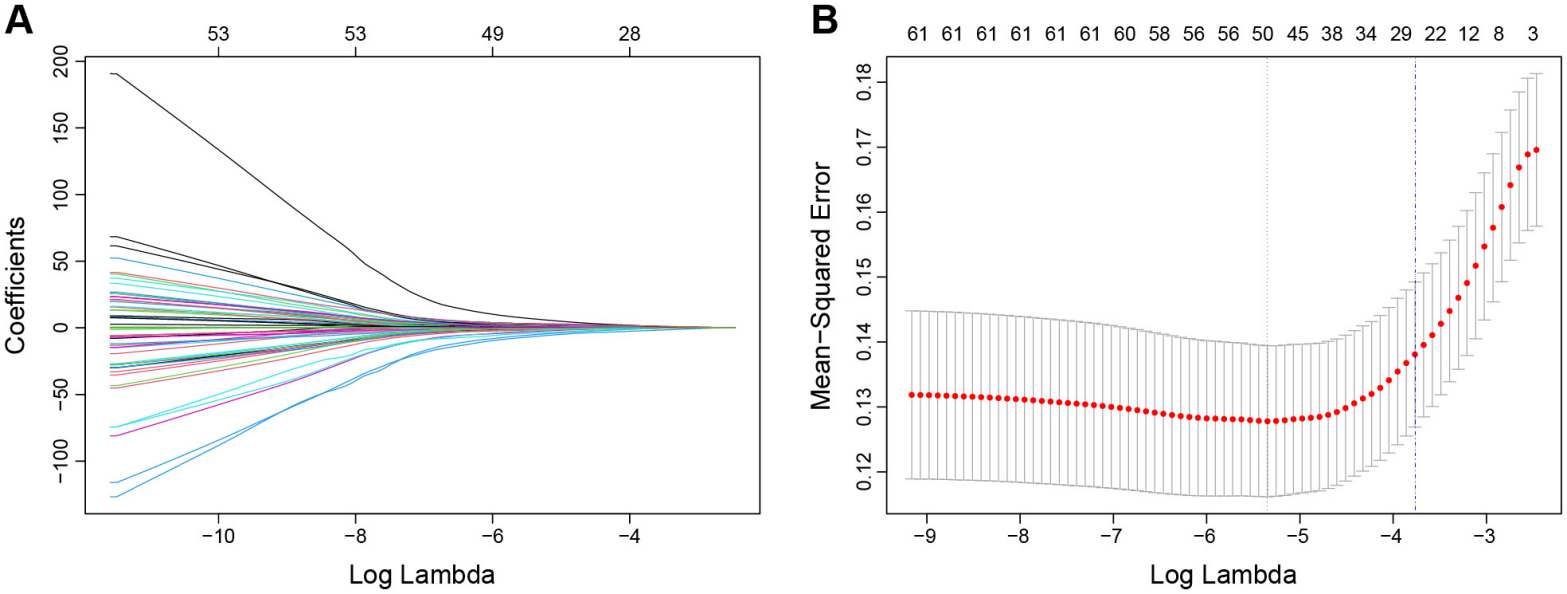
Selection of potential predictive factors using the least absolute shrinkage and selection operator (LASSO) regression. **(A)** LASSO coefficient profiles. **(B)** Ten-fold cross-validation for parameter selection (λ) in the LASSO logistic regression model. The blue vertical dashed line indicates the optimal value determined by the 1SE standard, and the black vertical dashed line represents the optimal value determined by the minimum standard. Based on 10-fold cross-validation and the 1SE standard, a λ value of 0.023 [log(λ) = −3.56] was selected, resulting in 26 non-zero coefficient variables.

**Figure 3:**
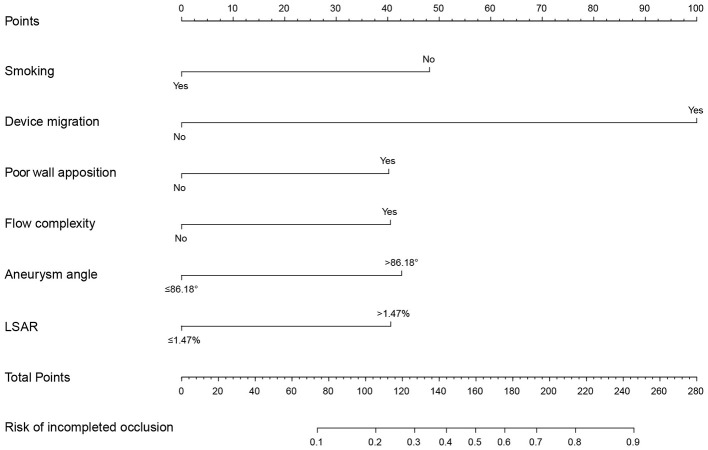
Hemodynamics-based nomogram predicting the risk of incomplete occlusion of intracranial aneurysms treated with pipeline embolization device.

In the training cohort, 26 potential predictors (nonzero coefficient variables) were selected using the LASSO regression model with the optimal lambda (λ) value determined at 1 SE (the optimal λ = 0.023) after 10-fold cross-validation (Figure 2). A backward stepwise multivariate logistic regression analysis was then performed on these variables, revealing that smoking (OR = 0.32, 95% CI 0.14–0.68, *p* = 0.005), flow complexity (OR = 3.03, 95% CI 1.58–5.89, *p* < 0.001), device migration (OR = 11.03, 95% CI 1.51–105.55, *p* = 0.021), poor wall apposition (OR = 3.21, 95%CI 1.37–7.53, *p* = 0.007), aneurysm angle (OR = 3.46, 95% CI 1.79–6.93, *p* < 0.001) and LSAR (OR = 2.78, 95% CI 1.46–5.50, *p* = 0.002) were independent predictors associated with ICO (Table 2). The correlation matrix of the above variables and variance inflation factor from the final multivariate regression model indicated that there was no multicollinearity among the variables (Supplementary Figure 2). A nomogram predicting ICO after PED treatment was developed based on the above independent predictors (Figure 3).

**Table 2:** Multivariate logistic regression analysis.

**Variables**	**β**	**OR (95% CI)**	***P***
Smoking	−1.145	0.32 (0.14–0.68)	0.005
Flow complexity	1.107	3.03 (1.58–5.89)	< 0.001
Device migration	2.401	11.03 (1.51–105.55)	0.021
Poor wall apposition	1.165	3.21 (1.37–7.53)	0.007
Aneurysm angle	1.240	3.46 (1.79–6.93)	< 0.001
LSAR	1.023	2.78 (1.46–5.50)	0.002

LSAR, low WSS area ratio.

### Validation and clinical use of the nomogram

**Figure 4:**
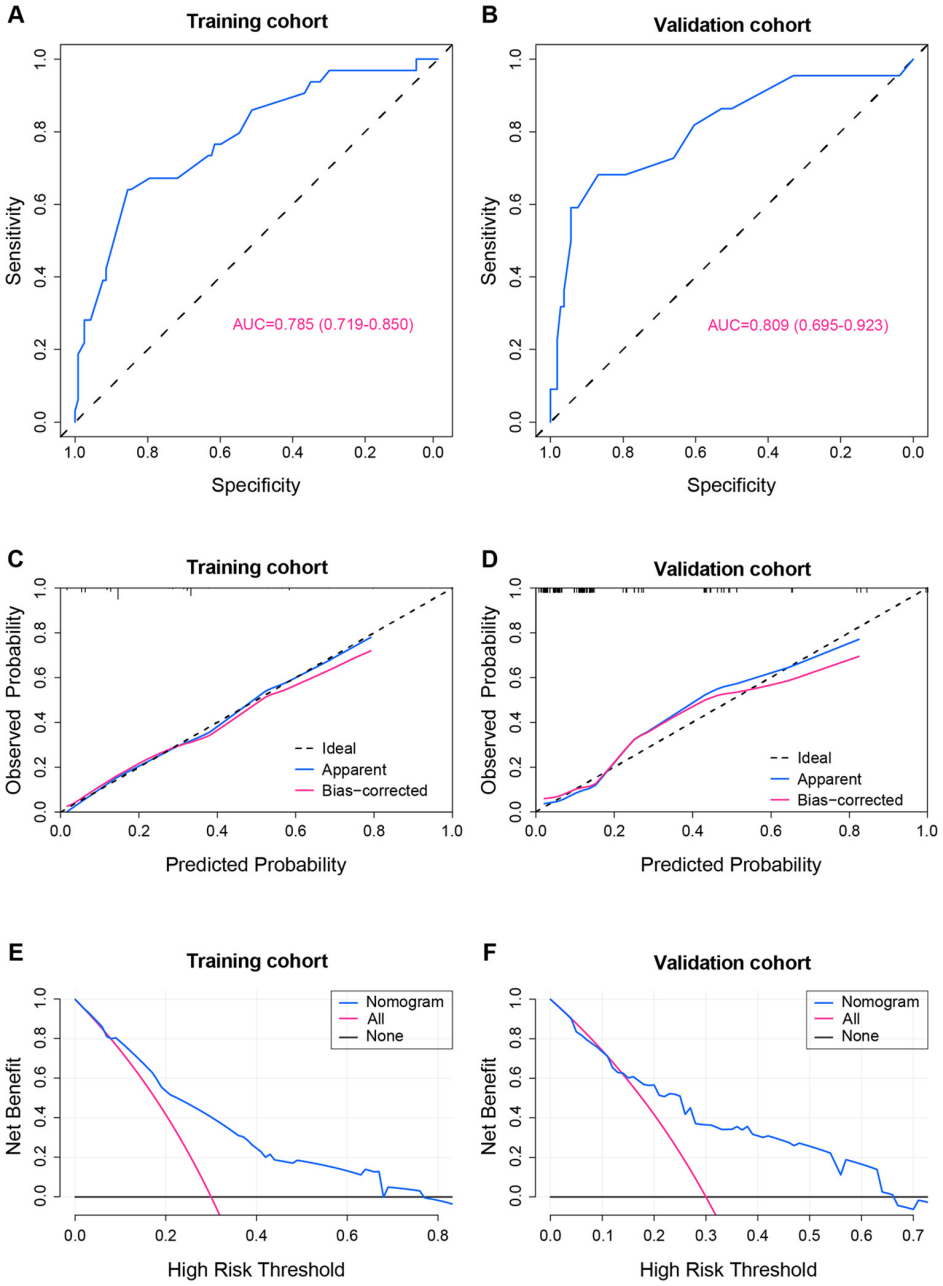
The receiver operating characteristic (ROC) curve, the calibration curve, and the decision curve analysis (DCA) of the nomogram model in each cohort. **(A)** ROC of the nomogram prediction in the training cohort. **(B)** ROC of the nomogram prediction in the validation cohort. **(C)** Calibration curve of the nomogram prediction in the training cohort. **(D)** Calibration curve of the nomogram prediction in the validation cohort. **(E)** DCA of the nomogram prediction in the training cohort. **(F)** DCA of the nomogram prediction in the validation cohort. AUC, area under the ROC curve.

The ROC curve suggested an AUC of 0.785 (95% CI 0.7719–0.850) in the training set (Figure 4A) and an AUC of 0.809 (95%CI 0.695–0.923) in the validation set (Figure 4B), indicating good discrimination of the nomogram in predicting the risk of ICO. The calibration curve showed strong consistency between prediction and observation in the training cohort (Figure 4C) and the validation cohort (Figure 4D). DCA revealed that our nomogram conferred a positive net benefit compared to the all-or-none within a threshold range of 0%−65% in both the training cohort and the validation cohort (Figures 4E, F).

**Figure 5:**
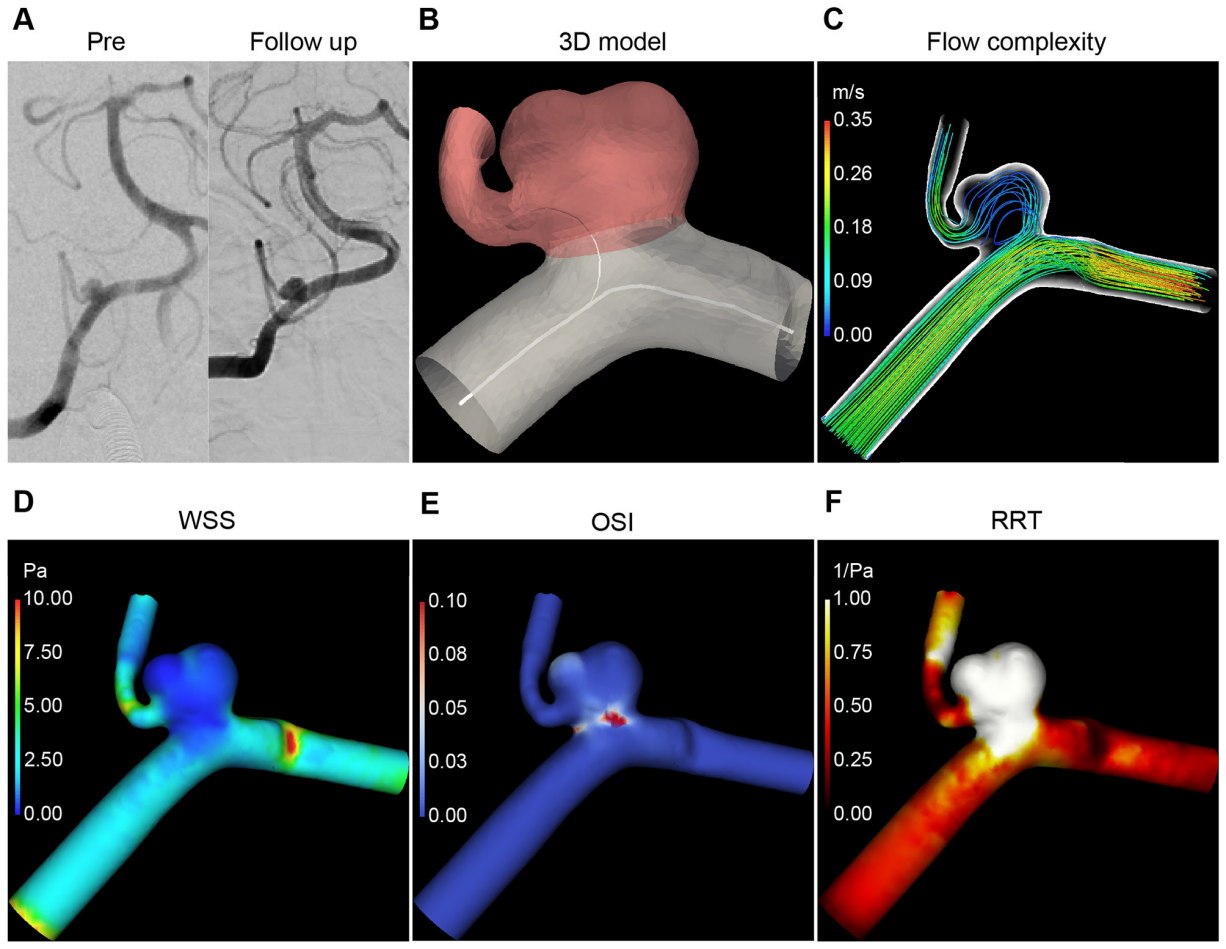
A representative case of an incompletely occluded intracranial aneurysm treated with a pipeline embolization device (PED). The patient had a right vertebral artery aneurysm, and follow-up angiography indicated persistent patency of the aneurysm 193 days after PED treatment. **(A)** Angiography images before PED treatment and 193 days after PED treatment. **(B)** 3D reconstruction model of the aneurysm-parent artery. **(C)** Flow pattern diagram (complex flow). Distribution map of mean wall shear stress (WSS) **(D)** mean oscillatory shear index (OSI) **(E)** and relative residence time (RRT) **(F)**. As shown in the hemodynamic schematic, this incompletely occluded aneurysm had a complex blood flow pattern and a large area of low WSS.

A representative case of ICO and its hemodynamic analysis were shown schematically in Figure 5.

## Discussion

Previous studies indicated that the 6-month CO rate of PED treatment for IAs range from 72 to 81.4% (1, 2, 4). In the whole cohort of our study, the occlusion rate of IAs was 79.8% with a median FU-DSA of 199 days. Six independent predictors of ICO were identified by further analysis and the first hemodynamics-based predicting nomogram for ICO of IAs treated with PED was developed and evaluated with excellent discrimination, calibration and clinical use.

### Smoking

The relationship between smoking and aneurysm CO has been previously described. In the multicenter series by Adeeb et al., non-smoking status was identified as an independent predictor of ICO, while Hanel et al. (8) demonstrated in the prospective study on embolization of intracranial aneurysms with the pipeline Device (PREMIER) cohort that smoking was a significant contributor to CO after PED implantation. We consistently found a correlation between smoking and IA occlusion after PED treatment. Adeeb et al. hypothesized that the prothrombotic state induced by smoking may enhance intra-aneurysmal thrombosis and subsequent aneurysm occlusion. Moreover, smoking contributes to atherosclerosis (18, 19) and decreases regional cerebral blood flow in the long term (20). This may explain the role of smoking in exacerbating altered hemodynamics and endothelial regenerative processes during vascular remodeling after PED treatment. This is further supported by the finding that smoking is an independent risk factor for in-stent stenosis, which is characterized by excessive endothelial proliferation in PED (21). A recent ICO risk scoring system for PED-treated aneurysms that included smoking as a negative predictor exhibited good performance as well (AUC = 0.71) (22).

### Device migration and poor wall apposition

Device migration after PED treatment is a rare complication with an incidence range of 3.2%−4.9% (12). Device migration and poor wall apposition would result in endoleak in the aneurysmal zone, which may alter the hemodynamic impact of PED treatment (23). In our study, the incidence of device migration and poor wall apposition was 2.2% (8/362) and 11.5% (49/426), respectively. In cases of endoleak, the aneurysm's neck and dome maintain high blood flow velocities, contributing to the persistence of aneurysm patency (7). Christoph et al. (24) utilized stereoscopic particle image velocimetry to demonstrate that stent malapposition, affected the reduction of intra-aneurysmal blood flow by PED and interfered with the oscillatory velocity index, thereby increasing the risk of ICO and in-stent thrombosis. Studies by Long et al. (7) and Zhang et al. (25) have demonstrated that stent malapposition leading to endoleak is an independent risk factor for ICO. In our study, the areas of poor wall apposition were not limited to the aneurysm neck. Therefore, we believe that any poor apposition of PED to the vessel wall may hinder the remodeling of the parent artery, based on the presence of endoleak, thereby affecting aneurysm occlusion.

Although device migration and poor wall apposition can be recognized in time, managing endoleak remains a subject of ongoing debate. Studies have shown that proactive management, such as balloon post-dilatation, repeated massage with the “J”-shaped microwire, and overlapping stenting, can improve stent apposition and reduce endoleak. However, these approaches may increase the incidence of related complications, including further device migration, thrombosis and vascular perforation, and increase patient radiation exposure (26, 27). Consequently, evaluating the risk of device migration and poor wall apposition, alongside identifying effective remedial strategies, is of considerable importance.

Several studies have suggested that adjunctive coiling, based on clinical experience, may act as a protective factor (6, 28, 29). However, LASSO variable selection and multivariate regression analysis did not reveal an independent effect of adjunctive coiling, which may be attributed to the loose packing and efforts aimed at reducing the risk of delayed rupture.

### Flow complexity

Flow complexity within an aneurysm sac is characterized by bifurcating or separated flow patterns, as well as multiple recirculation zones or vortex structures (16). These complex flow patterns disrupt the normal laminar flow, potentially creating a turbulent environment that impedes thrombosis formation and prevents effective aneurysm occlusion. While blood flow patterns can be visualized through software hemodynamic simulations, most computational fluid dynamics (CFD) studies assume laminar flow, which may not accurately reflect the complexities of real flow dynamics. As a result, the hemodynamic parameters derived from such simulations were not the completely true results of turbulence or complex flow.

The high-fidelity fluid structure interaction simulations of turbulent-like aneurysm flows by Souche et al. (30) revealed high-frequency narrowband wall vibrations, highlighting the relationship between blood flow patterns and mechanobiology. Previous studies have reported that high-frequency, high-energy turbulent-like flow, potentially coupled with wall vibrations, may contribute to the formation of IAs (31). Similarly, our study identified complex flow as a significant predictor of ICO in IAs. Therefore, realistic blood flow simulations, coupled with an understanding of their interaction with the structural mechanics of the aneurysm wall, which reflects the ability to withstand blood flow-induced stresses, provide valuable insights for advancing CFD techniques and furthering research in vascular pathology.

### Aneurysm angle from morphology

Previous studies have shown that aneurysm morphology factors, including aneurysm diameter, neck diameter, aspect ratio, and inflow angle, are significantly associated with ICO after PED treatment (6, 7, 32). Sunohara et al. (33) suggested that saccular aneurysms with an outer convex shape are more likely to experience ICO. Additionally, parent artery straightening could improve aneurysm occlusion rates (34). However, morphological effects inevitably manifest in changes of blood flow patterns and hemodynamic parameters, and focusing solely on morphological factors does not offer a complete understanding of the occlusion process. Chen et al. (35) found that a lower reduction in flow velocity throughout the aneurysm and neck before and after PED implantation is significantly associated with ICO. Considering this, the preoperative aneurysm angle (>86.18°) in our study, reflecting the relationship between the aneurysm neck and dome with little postoperative variation, significantly predicted ICO, likely due to its primary effect on blood flow within the aneurysm and the changes in flow velocity in the aneurysm and neck regions after PED implantation. The role of aneurysm angle may extend beyond its composite impact on hemodynamics, representing an independent morphological determinant in the statistical analysis. In contrast, other preoperative morphological parameters in our study—including branch incorporation [a predictor proposed in Hu et al.'s (29) machine learning model without hemodynamic parameters]—showed no correlation with ICO, precisely because the incorporation of flow patterns and hemodynamic parameters quantified and reflected the morphological effects. As illustrated in the representative case (Figure 5), although branch incorporation was present, this morphological factor ultimately resulted in flow complexity and high LSAR in the simulated hemodynamic environment, both of which contributed to ICO.

### LSAR

The interaction between blood flow and endothelial cells plays a vital role in the normal proliferation of the arterial wall. Low WSS and high LSAR have been widely studied and shown to be associated with a higher risk of aneurysm rupture (36–38). Slow or stagnant blood flow leads to an inflammatory response in the vessel wall (39). Both low WSS and high LSAR contribute to the progression of atherosclerosis (40) and can even lead to atherosclerotic changes in the aneurysm wall, suggesting significant damage to the aneurysm wall (41). In contrast to high WSS, which promoted anticoagulation, anti-inflammatory effects, proliferation and remodeling of the extracellular matrix, Morel et al. (42) found that low WSS downregulated the expression of cytoskeletal proteins in porcine arterial endothelial cells and upregulated extracellular matrix proteins, affecting endothelial remodeling of the aneurysm wall. Our findings similarly support these observations. High LSAR was significantly associated with aneurysm occlusion failure, likely due to endothelial cells' “inert” or “adverse” biological response to low friction. The influence of hemodynamic parameters is crucial for assessing aneurysm rupture, growth, and occlusion.

### Hemodynamics-based predictive nomogram

In our study, we combined LASSO for variable selection with multivariable logistic regression. This approach reduced the risk of underfitting associated with LASSO and alleviated the potential for overfitting in logistic regression. As a result, the hemodynamics-based predictive nomogram demonstrated strong predictive performance during validation. However, the predictive performance, discrimination and calibration of risk models do not capture the clinical consequences associated with specific levels of discrimination or calibration errors (43, 44). The main goal of a nomogram is to determine whether an individual needs additional treatment or care, based on their specific clinical needs (17). To demonstrate clinical utility, we used DCA, which showed positive net benefits within the threshold probability range of 0%−65%, indicating that the benefits of clinical interventions for patients would outweigh the potential harms within this range. More importantly, the indicators used in our nomogram are affordable and easily accessible. Both doctors and patients can use this user-friendly, relatively comprehensive scoring system to make individualized pre-treatment predictions for the ICO risk of IAs treated with PED, facilitating appropriate decisions and strategies, which is in line with the current trend of personalized medicine.

The ABC scoring system proposed by Ramirez-Velandia et al. (22) for assessing ICO risk (A = age, B = branch, C = cigarette smoking) with a discrimination of AUC = 0.71, was slightly lower than our model. While their scoring system is comparable, their model did not account for hemodynamics and had the inherent limitation of lacking extensive validation (only using ROC), making it far from ready for clinical implementation in individualized risk prediction. Additionally, Hammoud et al. (45) reported an optimal machine learning model with an accuracy of 89% in a cohort of 667 aneurysms. There are limitations to using machine learning for such predictions. Data augmentation and overfitting are significant challenges in machine learning, as models can predict results with high accuracy on their training and testing samples, but their ability to generalize beyond these datasets is limited.

### Limitation

First, this is a retrospective study, which inherently introduces selection bias in patient inclusion. Additionally, the sample was limited to the Chinese population and the eastern region, so the generalizability of the findings requires further validation. Third, due to selection bias by the operator, adjunctive coiling, a theoretically beneficial factor for CO, was found to be unrelated to aneurysm occlusion. Larger sample sizes are needed to minimize the impact of bias. Furthermore, the simplified assumptions in the blood simulation and CFD simulations have limitations, and advancements in simulation techniques are needed to better reflect real-world scenarios. Therefore, larger prospective studies, longer follow-up periods, and more comprehensive assessments are essential.

## Conclusion

By integrating preoperative DSA-based hemodynamic analysis, our multicenter study identified smoking, device migration, poor wall apposition, flow complexity, aneurysm angle, and LSAR as independent predictors of ICO in IAs after PED treatment. A nomogram was developed based on these factors with strong predictive performance for ICO risk of PED treatment in both internal and external validation, suggesting its potential as a valuable adjunct tool for clinicians to facilitate personalized treatment strategies.

## Data Availability

The raw data supporting the conclusions of this article will be made available by the authors, without undue reservation.
