# Supplementary material for: A multicenter hemodynamics–based nomogram predicting incomplete occlusion of intracranial aneurysms treated with pipeline embolization device
Source: Front Neurol. 2026 Feb 9;17:1756374. doi: 10.3389/fneur.2026.1756374 (PMC12926474; doi:10.3389/fneur.2026.1756374)
Supplement: Supplementary file 1 [file Image_1.pdf]

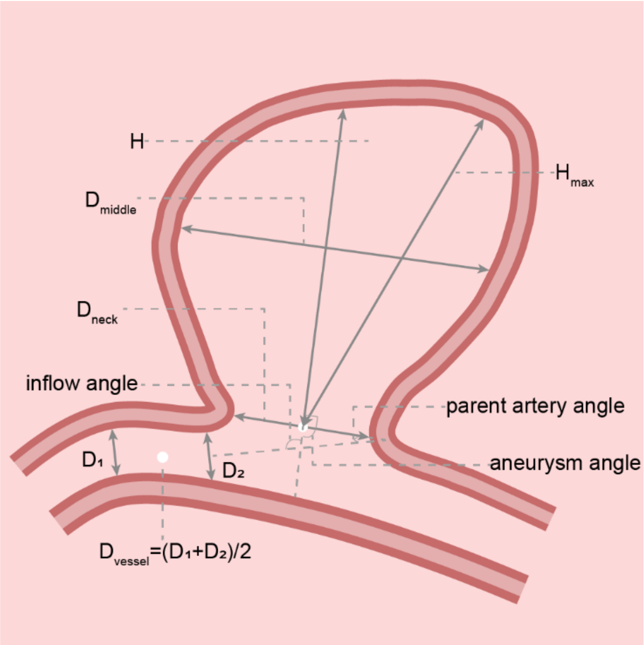

Supplementary Fig. 1. Schematic diagram of morphological parameters. H_{max}: maximum height of the aneurysm; D_{middle}: middle diameter of the aneurysm; D_{neck}: neck diameter of the aneurysm; D_{vessel}: diameter of the parent artery; H: vertical height of the aneurysm.

A

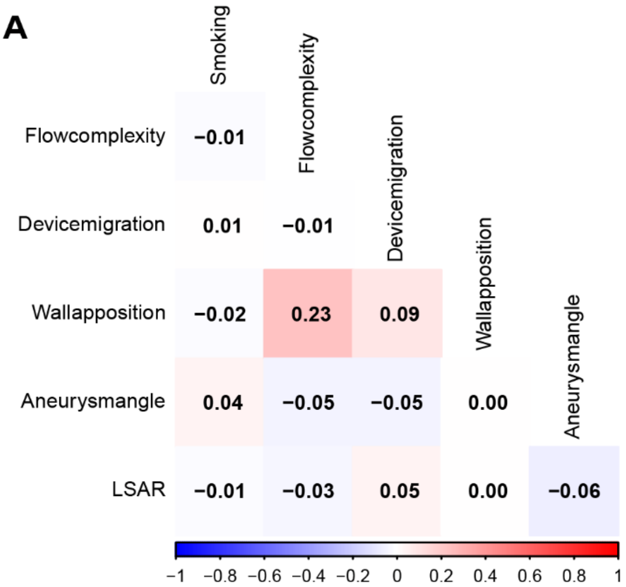

B

Variable	VIF
Smoking	1.043
Device migration	1.092
Poor wall apposition	1.102
Flow complexity	1.116
Aneurysm angle	1.153
LSAR	1.100

Supplementary Fig. 2. Multicollinearity assessement with correlation matrices and the variance inflation factor (VIF). A, Correlation matrix of the nomogram model. B, VIF analysis of variables in multivariable regression model. LSAR, low WSS area ratio.
